# Postnatal growth and gut microbiota development influenced early childhood growth in preterm infants

**DOI:** 10.3389/fped.2022.850629

**Published:** 2022-08-09

**Authors:** Jocelyne S. Tadros, Amelia Llerena, Anujit Sarkar, Reynold Johnson, Elizabeth M. Miller, Heewon L. Gray, Thao T. B. Ho

**Affiliations:** ^1^Department of Pediatrics, Morsani College of Medicine, University of South Florida, Tampa, FL, United States; ^2^College of Public Health, University of South Florida, Tampa, FL, United States; ^3^Department of Anthropology, College of Arts and Sciences, University of South Florida, Tampa, FL, United States

**Keywords:** preterm infants, gut microbiome, catch-up growth, nutrition, childhood BMI

## Abstract

**Background:**

Preterm infants are at high risk for growth failure and childhood weight problems due to the disruption of normal intrauterine growth and nutrition. Early nutritional support and microbiome acquisition can play an important role in childhood growth.

**Objective:**

Our study examined potential postnatal indicators, including gut bacterial compositions, macronutrients, and catch-up growth, of growth pattern from infancy into early childhood.

**Methods:**

This is a retrospective study of preterm infants born < 35 weeks who were followed up in the university complex care clinic from 2012–2018. Weight and length z-scores at birth, 1, 2, 4, 6, 12 and 15 months, and body mass index (BMI) and length z-scores from 2 to 5 years of age were collected. Catch-up growths were calculated by changes in z-scores and divided into early (birth-4 months) and late (4–18 months). Postnatal nutritional data and fecal samples were collected. Fecal microbiome data obtained from 16S RNA V4 sequencing was analyzed against clinical and growth data using a regression model.

**Results:**

160 infants included in the final analysis had birth weight and gestational age of 1,149 ± 496 grams and 28 ± 3 weeks. Early weight gain positively correlated with length z-scores but not with BMI at 2 years of age. BMI at 2 years of age strongly correlated with BMI at 3, 4, and 5 years of age. Postnatal abundance of Gammaproteobacteria was negatively associated with early growth while Bacteroides and Lactobacillus were positively associated with childhood BMI.

**Conclusion:**

Our findings suggest that optimal postnatal nutrition promoted early catch-up growth in weight as well as improved linear growth without influence on childhood BMI. Postnatal gut microbial colonization, which is a modifiable factor, was associated with childhood growth in preterm infants.

## Introduction

Due to prematurity and compromised intrauterine environment, preterm infants commonly experience growth deficit, which often presents as a low birth weight for age and/or growth failure occurring shortly after birth. Combating this growth deficit is important for the long-term outcomes of these infants. The trajectory of post-natal growth of preterm infants correlates with childhood growth patterns and cognitive development (1). Insufficient growth can negatively impact immune, gastrointestinal, and neurological development. Further, some studies have demonstrated that timing of catch-up growth is critical for optimal development and missed opportunities may not be reversible (2). Catch-up growth is a phase of rapid linear growth that allows an infant to accelerate toward and resume their pre-restriction growth curve. Although catch-up growth could help preterm infants reach their ideal anthropometrics, many preterm infants experience post-natal growth failure. Understanding the relationships among early nutritional support, gut microbiota, and childhood growth as well as identifying predictors of childhood growth assist clinicians to optimize early postnatal nutritional care.

To assess growth from birth into late adolescence and early adulthood, the latest World Health Organization (WHO) guidelines provide seamless standards and references (3). Preterm growth is measured using the Fenton Growth charts, which transition to the WHO Childhood Growth Charts (4, 5). Changes in growth can be assessed through change in z scores to indicate height and weight changes in reference to the WHO Childhood Growth Standards. Body mass index (BMI), a simple index of weight-for-height, is commonly used to classify overweight and obesity. To gain a better understanding of the growth trajectory during infancy, growth can be classified as early from birth—4 months of age, and late, from 4–18 months of age (6, 7).

The postnatal growth failure that preterm infants often experience has been linked to the immaturity of gut microbiota (8, 9). The microbiota of infants experiencing growth failure has been previously characterized by low diversity, increased abundance of *Staphylococcaceae* and *Enterobacteriaceae*, and lack of *Veillonella* as compared to infants with appropriate growth (8). Nutrition in early infancy plays an important role in both growth and bacterial colonization of the gut (10). The composition of the microbiota is vital in the breakdown of food and utilization of nutrients to support growth during infancy. The early bacterial colonizers of the infant gut help regulate future changes throughout childhood (11). However, there is no consensus on which bacterial taxa predispose these infants to growth deficit. Determining which bacterial taxa aid and inhibit catch-up growth in preterm infants is important to optimize healthy childhood anthropometrics.

Few studies have investigated the role early microbial acquisition on childhood growth among preterm infants. Thus far, early microbiome volatility, potentially indicating greater bacterial diversity, correlates with improved growth in length at ages 2 through 4 (12). Identifying factors that can help combat post-natal growth deficit of preterm infants is critical to their later development. This study aims to examine the relationship among early postnatal nutrition and microbiome, infancy growth, and early childhood BMI. These relationships may identify early life predictors of optimal childhood growth for preterm infants.

## Materials and methods

### Patient selection and data collection

This was a retrospective study of infants who were born with birth gestational age (GA) < 35 weeks, admitted to the level 3 academic neonatal intensive care unit (NICU) at Tampa General Hospital between January 1, 2012 to December 31, 2018, and had at least one well child visit between 2 and 5 years of age at the University of South Florida (USF) Complex Care Clinic. Exclusion criteria were congenital anomalies or chromosomal disorders. The study was approved by the USF Institutional Review Board.

The data were collected from both inpatient and outpatient electronic medical records of the infants on NICU admission and outpatient care. The collected demographic and clinical information included birth weight (BW), birth gestational age (GA), gender, ethnicity, race, maternal age, intrauterine growth restriction (IUGR) and small for gestational age (SGA) status, maternal comorbidities (hypertension, preeclampsia, diabetes mellitus), maternal BMI, multiple gestations, NICU length of stay (LOS), grade 3 or 4 intraventricular hemorrhage (IVH), necrotizing enterocolitis (NEC), and chronic lung disease (CLD) (defined as oxygen requirement at 36 weeks corrected gestational age). We collected detailed information on nutritional supports during their NICU admissions and their first 5 years of life, such as the daily caloric intake, days to full feeds, and macronutrient supplementations—protein and medium-chain triglycerides (MCT). The standard caloric concentration is 24 kcal/oz for preterm infants born < 32 weeks and daily caloric concentration > 24 kcal/oz was considered high caloric concentration.

### Growth curves and catch-up growth

We recorded each infant’s anthropometric data from birth to 5 years of age including weekly weight and length along with their z-scores on the revised Fenton growth charts up to 2 months chronological age and on WHO growth charts thereafter, and their BMI at ages 2–5 years. For children under 5 years of age, overweight is defined as BMI greater than 1 standard deviations and obesity is defined as BMI greater than 2 standard deviations above the WHO Child Growth Standards median (5). In our study, early catch-up growth was defined as from birth to 4 months, often during NICU admission, and the late catch-up growth as from 4 to 18 months, often post NICU admission.

### Stool collection

A number of the above infants (*n* = 34) had weekly fecal samples collected during their NICU admission in an observational study approved by the University of South Florida Institutional Review Board. The inclusion criteria for this cohort are birth weight < 1,500 g or birth gestational age < 33 weeks. *Infant fecal samples were collected weekly from diapers and stored immediately at −80*°*C until processing for DNA extraction for microbiome analysis*. Stool samples collected at < 28 days and at 36-week corrected Ga (cGA) or prior to discharge were included in the final analysis. Stool samples were categorized by postnatal age at collection: during the first 14 days of life, 14–28 days of life, and at 36-week cGA.

### DNA extraction and 16s rRNA gene sequencing library preparation

Total DNA was extracted from thawed fecal samples using QIAamp PowerFecal DNA extraction kit (Qiagen, Carlsbad, CA, United States) following the manufacturer’s instructions. The amount of DNA was measured with the Qubit 3.0 fluorometer (Life technologies, Carlsbad, CA, United States). The bacterial 16S rRNA V4 gene region was amplified by polymerase chain reaction using 515F and 806R primers (IDT, Coralville, IA, United States). The gene was then sequenced using the 2 bp × 300 bp chemistry in a paired-end manner on the Illumina Miseq platform (Illumina, San Diego, CA, United States).

### 16S rRNA analysis

All the raw demultiplexed fastq files were imported in R version 4.1.0 and were analyzed by the dada2 package v1.10.1 (13). Briefly, the forward and reverse reads were trimmed to 270 and 220 bases and the expected error for forward and reverse was set to 2 and 4 respectively. All ambiguous bases were discarded and the phiX genome was removed. The amplicon sequence variants (ASVs) were identified separately for the forward and reverse sequences and were merged subsequently. All identified chimeras were removed and the filtered ASVs were matched against the Silva v132 database to obtain their bacterial classification. Finally, the bacterial distribution table for all samples were obtained and a phyloseq (14) object was created for further analysis.

### Statistical analysis

Continuous variables were described by mean and standard deviations (SD) or median and interquartile range (IQR) while categorical variables were described by percentages. The association between categorical variables was evaluated by the Fisher’s exact test. For continuous variables, independent samples *t*-tests and Mann–Whitney *U* tests were applied for normally distributed and skewed data, respectively. Because many comorbidities, such as CLD and NEC, occurred more frequently in infants born at lower birth GA and weight, we controlled for birth GA and weight in our analysis. The 95% confidence intervals were used to describe the precision of the estimates. The two-tailed statistical tests were considered significant at *p* < 0.05. All non-microbiome analyses were performed using IBM SPSS statistical software package (IBM Corp., IBM SPSS Statistics, Version 27.0 Armonk, NY: IBM Corp.). We also used SPSS to calculate the power with a linear regression model and two controlling factors (birth GA and weight) to predict childhood growth measurement with postnatal microbiome. A sample size of 34 subjects will detect an effect size of 0.3 at 0.871 power with a significance alpha level of 0.05. For Pearson correlation between BMI datasets, a sample size of 24 will detect a correlation coefficient of 0.6 at 0.901 power and alpha level of 0.05. The associations for BMI, length z-scores and weight z-scores with the microbiome were tested separately employing optimal microbiome-based association test (OMiAT) in R (15). OMiAT performs microbiome regression-based kernel association tests (MiRKATs) based on various distance measures. Here, all the associations were tested with 5,000 permutations after correcting for gestational weight, gender, current weight, maximum calorie intake, discharge weight, and maximum caloric intake. To determine whether the individual ASVs were associated with the phenotypes, the Microbiome Comprehensive Association Mapping (MiCAM) function implemented in the OMiAT package was used with 5,000 permutations. For all tests, only those ASVs were considered if they had a minimum abundance of 0.01% in at least one sample, and the ASV was observed in a minimum of 10% of all the samples. The associations with a *p*-value ≤ 0.05 are reported here.

## Results

### Maternal and infant characteristics

We screened 445 infants who had at least one well child visit at the USF complex care clinic. We included 160 infants who met our inclusion and exclusion criteria and 34 out of 160 infants had stool samples collected for microbiome analysis (Supplementary Figure 1). Their mean BW and GA were 1,149 ± 496 grams and 28 ± 3 weeks, respectively, with 81% (*n* = 130) very-low-birth-weight (VLBW, < 1,500 g) infants and 12% (*n* = 19) SGA (Table 1).

**TABLE 1:** Patient demographics and characteristics.

Characteristic	*N* = 160
Gestational age, weeks (mean, SD)	28 (3)
Birth weight, grams (mean, SD)	1,149 (496)
IUGR, n (%)	24 (15%)
Small for gestational age, n (%)	19 (12%)
Multiple birth, n (%)	42 (26%)
Male, n (%)	85 (53%)
Hispanic ethnicity, n (%)	32 (20%)
Race, n (%) Black White Other	55 (35%) 87 (54%) 18 (11%)
Maternal hypertension, n (%)	35 (22%)
Maternal preeclampsia, n (%)	47 (29%)
Maternal diabetes mellitus, n (%)	25 (16%)
Maternal BMI, mean (SD)	30 (8)
CLD, n (%)	36 (22.5%)
NEC, n (%)	4 (2.5%)
IVH, n (%)	8 (5%)
Supplementation, n (%) None Protein MCT Protein and MCT	94 (59%) 38 (23%) 6 (4%) 22 (14%)
Feeding type at discharge, n (%) Maternal breast milk only Formula only Mixed feeding types	40 (25%) 82 (51%) 38 (24%)
Length of stay, days, (mean, SD)	80 (48)

IUGR, intrauterine growth restriction; BMI, body mass index; CLD, chronic lung disease; IVH, intraventricular hemorrhage; MCT, medium chain triglycerides.

### Early childhood growth

**FIGURE 1:**
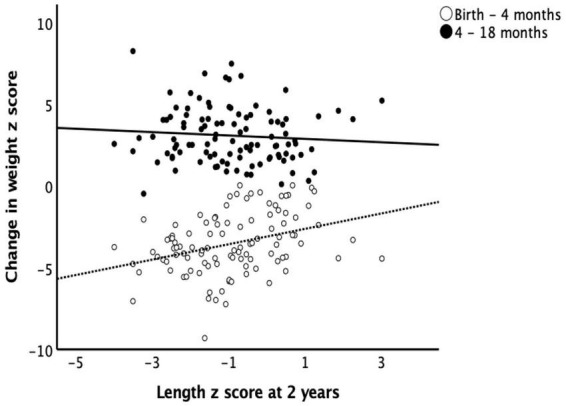
Length at 2 years and catch-up growth. Early catch-up growth, defined as change in weight z score from birth to 4 months of age, had a positive correlation (*r* = 0.302, *p* = 0.001) while late catch-up growth, defined as change in weight z score from 4 to 18 months of age, had a negative correlation (*r* = –0.317, *p* = 0.001) with length z scores at 2 years of age.

The mean changes in weight z scores from birth to 4 months and from 4 to 18 months were −3.53 ± 1.85 and 4.07 ± 2.29. The mean changes in length z scores from birth to 4 months and from 4 to 18 months were −3.39 ± 1.87 and 3.14 ± 1.72 respectively. Length z scores at 2 years correlated positively with the changes in weight z scores from birth to 4 months and negatively with that change from 4 to 18 months (*r* = 0.302, *p* = 0.001 and *r* = −0.317, *p* = 0.001 respectively) (Figure 1).

### Childhood BMI

**FIGURE 2:**
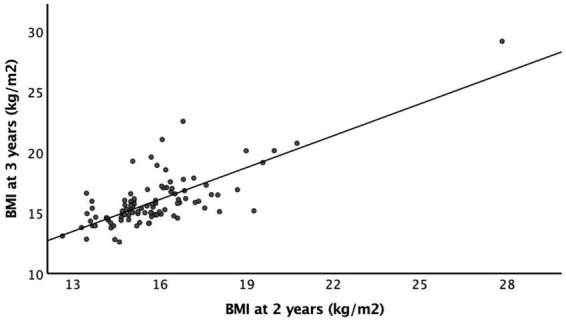
Body mass index as 2 and 3 years of age. There is a strong positive correlation between BMI at 2 years of age and BMI at 3 years of age (*r* = 0.753, *p* < 0.001).

A greater number of infants had a recorded BMI at 2 and 3 years than at 4 and 5 years. Of the 160 infants, the numbers of infants that had a documented BMI were: 93% (*n* = 149) at 2 years of age, 65% (*n* = 105) at 3 years, 25% (*n* = 40) at 4 years, and 15% (*n* = 24) at 5 years of age. At 2 years, the mean BMI was 16.1 ± 1.8 kg/m^2^, 11% (*n* = 17) of infants were overweight (BMI > 1 standard deviation above the mean), and 6% (*n* = 10) infants were obese (BMI > 2 standard deviations above the mean) based on the WHO definitions. Most infants (*n* = 114, 71%) had a BMI within the healthy range at 2 years of age (Table 2). The mean BMI was 15.8 ± 2.2 kg/m^2^ at 3 years of age, 16.1 ± 3.5 kg/m^2^ at 4 years of age, and 16.4 ± 4.1 kg/m^2^ at 5 years of age. BMIs at 2 years of age positively correlated with BMIs at 3, 4, and 5 years of age (*r* = 0.753, *p* < 0.001; *r* = 0.652, *p* < 0.001; and *r* = 0.836, *p* < 0.001 respectively) (Figure 2). We used BMI at 2 years as the representative of the childhood BMI in the following analyses.

**TABLE 2:** Anthropometrics data.

Characteristic	*N* = 160
Body Mass Index (BMI)*, n (%) Failure to Thrive Underweight Healthy Overweight Obese	7 (4%) 12 (8%) 114 (71%) 17 (11%) 10 (6%)
**Catch-up Growth, mean (SD)** Mean change in weight z score 0–4 months 4–18 months Mean change in length z score 0–4 months 4–18 months	−3.53 ± 1.85 (*n* = 129, 81%) 4.07 ± 2.29 (*n* = 105, 66%) −3.39 ± 1.87 (*n* = 130, 81%) 3.14 ± 1.72 (*n* = 98, 61%)

*WHO definitions: Failure to Thrive BMI is under 13.5 for girls and under 13.9 for boys.

Underweight BMI is 13.6–14.4 for girls and 14.0–14.8 for boys.

Healthy BMI is from 14.5–17.1 for girls and from 14.9–17.3 for boys.

Overweight BMI is from 17.2–18.4 for girls and from 17.5–18.6 for boys.

Obese BMI is over 18.5 for girls and 18.7 for boys.

### Early childhood growth and BMI

**FIGURE 3:**
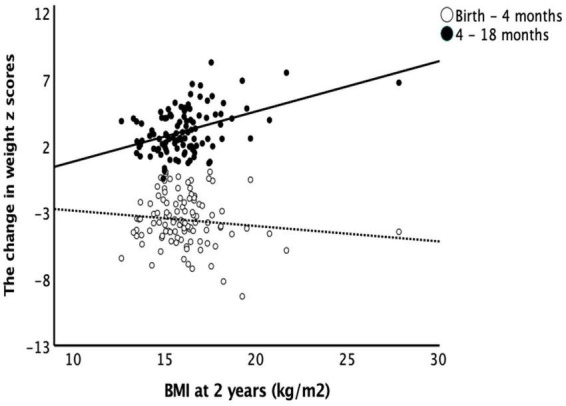
Body mass index at 2 years and catch-up growth. Early catch-up growth, defined as change in weight z score from birth to 4 months of age, did not correlate with BMI at 2 years of age (*r* = –0.103, *p* = 0.256). Late catch-up growth, defined as change in weight z scores from 4 to 18 months of age, had a positive correlation with BMI at 2 years of age (*r* = 0.417, *p* = 0.001).

The change in weight z scores from birth to 4 months did not correlate with BMI at 2 years with and without controlling for birth GA and BW. The change in weight z scores from 4 to 18 months positively correlated with BMI at 2 years of age (*r* = 0.417, *p* = 0.001) and the correlation remains significant with controlling for birth GA and BW (*r* = 0.469, *p* < 0.001). BMI at 2 years of age also had positive correlations with the change in length z scores from 4 to 18 months of age (*r* = 0.31, *p* = 0.002) but not with the change in length z scores from birth to 4 months (Figure 3).

### NICU nutrition in relation to postnatal microbiome development and childhood growth

About half (49%) of the infants (*n* = 78) were fed human milk (including donor breast milk, mother’s own breast milk, or a combination of the two) and 25% (*n* = 40) were exclusively breastfed at discharge (Table 1). Within the population, 41% received at least one macronutrient supplementation with 14% (*n* = 22) receiving both protein and MCT. About a third (31%) received high caloric concentration (>24 kcal/oz) during NICU admission, 31% received high daily caloric intake (≥140 kcal/kg/day), and 39% were discharged home with ≥24 kcal/oz feeding.

Length z scores at 2 years had a negative correlation with caloric supports measured as maximum caloric density (calories/ounce) and maximum caloric intake (calories/kg/day) during NICU (*r* = −0.357, *p* < 0.001 and *r* = −0.323, *p* < 0.001 respectively) and caloric density (calories/oz) of discharge feed (*r* = −0.302, *p* < 0.001). There was no association between nutritional supports (macronutrient supplementation and caloric density) and childhood BMI. We did not find any significant association between postnatal microbiome development and feeding types, caloric supports, or macronutrient supplementation.

### Postnatal microbiome development and early childhood growth

#### Birth to 4 month growth

Using the stated regression analysis, the change in length z scores from birth to 4 months positively associated with the fecal abundances of *Streptococcus*, *Veillonella*, and *Haemophilus* (*p* = 0.031, *p* = 0.044, and *p* = 0.002, respectively) from 0 to 14 days of age, negatively associated with the abundance of *Proteus* (*p* = 0.04) from 14 to 28 days of age and was not significantly associated with microbiome composition after 28 days of life.

**FIGURE 4:**
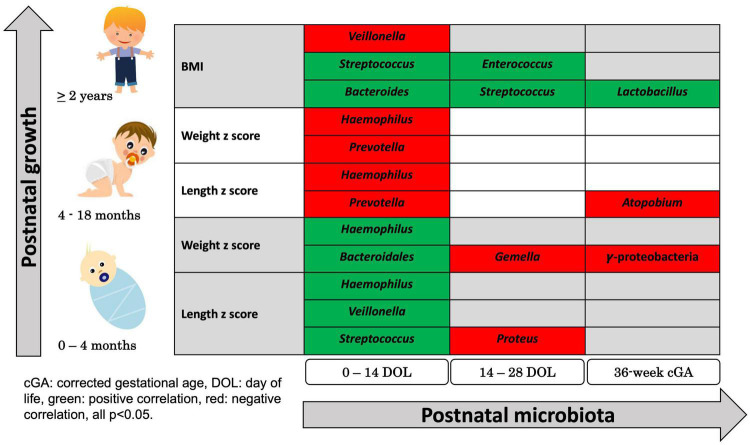
Summary of Significant Correlations Between Postnatal microbial abundance and early childhood growth. Using regression-based association tests between individual bacterial classification and growth, bacterial abundances 0–14 days, 14–28 days, and at 36 weeks corrected gestational age were associated with childhood growth measured by change in z-scores in weight and in length during early infancy, z-scores in weight and in length during late infancy, and BMI at 2 years of age. Green colored bacterial names had positive correlation with growth and red colored bacterial names had negative correlation with growth (all *p* < 0.05).

The change in weight z scores from birth to 4 months positively associated with abundance of *Bacteroidales* and *Haemophilus* (*p* = 0.042 and *p* = 0.024, respectively) from 0 to 14 days of age, negatively associated with abundances of *Gemella* (*p* = 0.032) from 14 to 28 days of age, and negatively associated with abundance of Gammaproteobacteria, a class of Proteobacteria (*p* = 0.018) at 36 weeks cGA (Figure 4).

#### 4–18 month growth

The change in length and weight z scores from 4–18 months were negatively associated with the abundances of *Prevotella* and *Haemophilus* (*p* = 0.034 and *p* = 0.031, respectively, for length and *p* = 0.02 and *p* = 0.029, respectively, for weight) from 0 to 14 days of age. The length z scores negatively associated with abundance of *Atopobium* (*p* = 0.027) after 28 days of age (Figure 4).

#### BMI at 2 years of age

BMIs at 2 years of age positively associated with abundance of *Bacteroides* and *Streptococcus* (*p* = 0.037 and *p* = 0.003, respectively) and negatively correlated with abundance of *Veillonella* (*p* = 0.018) from 0 to 14 days of age. BMI at 2 years of age was positively associated with abundance of *Streptococcus* and *Enterococcus* (*p* = 0.021 and *p* = 0.018, respectively) from 14 to 28 days of age and was positively associated with abundance of *Lactobacillus* (*p* = 0.035) at 36-week cGA (Figure 4).

## Discussion

Our study examined the nutritional supports and gut microbiota development of preterm infants in the NICU as predictors of early childhood anthropometrics. The data showed that optimized early postnatal growth in the NICU improved childhood linear growth without an impact on childhood BMI. Infants who required higher caloric intake to improve growth in NICU were at higher risk for poor linear growth at 2 years of age. The NICU microbiome was associated with anthropometrics from birth to 2 years. The trajectory of childhood BMI can be identified as early as 2 years of age and influenced by late infancy growth but not by early infancy growth. These findings support the benefits of early postnatal catch-up growth and the role of gut microbiota acquisition on childhood growth in preterm infants.

The importance of optimizing nutrition and growth in preterm infants in the NICU to improve long term neurodevelopmental outcomes has been well studied and documented (16). Our results show that optimal growth in the NICU establishes desired childhood growth patterns in preterm infants. Preterm growth can be improved through nutritional supplementation and increased caloric density (17) but some fear aggressive caloric supports in NICU can lead to childhood obesity (6, 7, 18). Ideally, nutritional intervention supports linear growth and lean body mass more so than fat deposition to achieve a healthy BMI (18). In our study, requiring increased caloric density and/or daily caloric intake using protein and MCT supplementations during the postnatal period in the NICU was an indicator of poorer growth in length at 2 years. However, the resulting early catch-up growth correlated with better linear growth but not associated with BMI ay 2 years. Our findings are in line with the findings of Regan et al. (19), which showed no significant association between macronutrient or energy intake and insulin sensitivity in preterm infants at 4–10 years of age. These findings suggest that using macronutrient and caloric supplementations to improve growth in the NICU may benefit metabolic health into childhood (20). Raaijmakers et al. (21) found that catch-up growth in infancy of low-birth-weight infants correlated with lower body fat in young adolescence. Other studies note concerns that accelerated growth velocity may predispose children to later obesity (22). Lapillonne et al. (23) discusses how growth in infancy and childhood can predispose patients to metabolic syndrome and hypertension. Our study showed that early catch-up growth in NICU did not appear to influence childhood BMI.

An early predictor of healthy childhood body weight is important for interventions to prevent the long-term negative health impacts from overweight and obesity. Even though early infancy growth did not predict childhood BMI, late infancy growth did. Weight gain during early infancy from birth to 4 months, often while the preterm infant was in the NICU, did not correlate with BMI at 2 years even after controlling for birth GA and BW. Whereas weight gain during late infancy from 4 to 18 months, usually after the preterm infant was discharged from the NICU, positively correlated with BMI at 2 years of age; this correlation remained significant after controlling for birth GA and BW. In our population, 2-year BMI predicted BMI through 5 years of age, which is consistent with the finding by Pryor et al. (24). This implies outpatient nutrition education for parents of preterm infants may be a high impact intervention to promote healthy childhood body weight.

Our study also found infant gut microbiota acquisition to be associated with childhood growth. Bacterial exposure from delivery mode, breast feeding, and hospitalization greatly influence the infant microbiome in the postnatal stage (9, 25). Our group and collaborators have shown the impact of perinatal factors, genetic relatedness, and NICU diet on postnatal microbiota development as well as the link between microbiota and growth during NICU admission (12, 26–30). In this study, we focused on the association between postnatal microbiota development and childhood growth into preschool age in preterm infants. We found links among bacterial abundance, catch-up growth, and childhood BMI; further supporting previous data showing that specific bacterial abundances correlate with growth deviance in childhood (8, 11). During NICU stay, weight gain negatively correlated with the abundance of Gammaproteobacteria. *Enterobacteriaceae*, a member of the Gammaproteobacteria taxa, has been implicated in delayed growth (27). These bacteria utilize metabolic pathways that produce less adenosine 5′-triphosphate (ATP) compared to beneficial *Bifidobacteria* and produce pro-inflammatory lipopolysaccharides (LPS) which decreases nutrient absorption in the colon (27). Our data also showed that decreased abundance of *Prevotella* correlates with higher childhood BMI and increased late infancy growth in weight and in height. Although *Prevotella* is classified as commensal bacteria, there are emerging studies suggesting its role in inflammatory disease and obesity. As most of the infants in our study were classified in the healthy BMI range at 2 years of age (71%), our finding most likely supports that decreased abundance of *Prevotella* limits inflammatory mechanisms involved in obesity development (31). Additionally, the data demonstrated that abundance of *Bacteroides* positively correlating with childhood BMI and infancy growth. Similar to our findings, Indiani et al. (32) found that the abundance of *Bacteroides* positively correlates with childhood BMI as well as childhood obesity. Due to the small number of children in our study classified as obese, our conclusions on the role of microbiome abundances in preterm infants on childhood obesity are limited. Instead, our results indicate that neonatal microbiota acquisition in preterm infants correlates with growth measures throughout infancy and childhood.

Nutrition in early infancy has been shown to impact the growth of preterm infants as well as influence bacterial diversity (10, 11). Breast milk in early infancy can stimulate beneficial bacteria such as *Bacteroides fragilis*, *Bifidobacteria infantis*, and *Lactobacillus acidophilus* through the production of immunoglobulin A (IgA), activation of regulatory T cells, and promotion of anti-inflammatory mediators (10). In a longitudinal study of term and preterm infants, the main factor in microbiome development during the first year of life was the receipt of breast milk, which was significantly associated with *Bifidobacterium* species throughout the first 3 years of life (33). In our study, about half (49%) of the infants were fed breast milk (including donor breast milk, mother’s own breast milk, or a combination of the two) and 25% were exclusively breastfed at discharge. Our microbiome pattern may not reflect the colonization and establishment of gut microbiota in a population with higher exposure to human milk or mother’s own milk. Although we did not find any significant associations between postnatal microbiome development and nutritional supports, a larger sample for microbiome analysis would be beneficial. Further study is needed to understand the relationship between NICU nutrition and the microbiome in early infancy.

Postnatal bacterial colonization and growth are early influences that impact childhood growth for preterm infants. The strength of this study was a cohort of preterm infants who were treated in a single center and followed up at one NICU follow-up clinic. The subjects were all under the same standards of care, feeding protocols, and monitoring of growth parameters. The longitudinal data and stool collections strengthened the findings of this study, but microbiome analysis for a greater number of the infants would be more desirable. In the future, a larger sample size should be evaluated to determine trends in microbiome diversity and childhood BMI.

## Conclusion

In preterm infants, maximizing childhood health outcomes begins with early interventions and exposures. Our study shows that early catch-up growth and microbiome establishment are important for supporting optimal childhood growth in preterm infants. Further, growth parameters in infancy can predict metabolic health into childhood. These findings offer an opportunity for clinicians and caregivers to cultivate positive health outcomes for preterm infants. The extended effects of early NICU interventions may be amplified by continued comprehensive care outside the NICU, involving NICU follow up clinic, primary care providers, and caregivers.

## Data availability statement

The datasets presented in this study can be found in online repositories. The names of the repository/repositories and accession number(s) can be found below: https://www.ncbi.nlm.nih.gov/, SRP171050.

## Ethics statement

The studies involving human participants were reviewed and approved by University of South Florida IRB. Written informed consent to participate in this study was provided by the participants’ legal guardian/next of kin.

## Author contributions

JT, EM, HG, and TH designed research. JT, RJ, AL, and TH conducted research. TH and AS analyzed data. JT, AL, AS, and TH prepared the manuscript. JT, AL, and TH had primary responsibility for final content. All authors read and approved the final manuscript.
