# Supplementary figures and images for: Postnatal growth and gut microbiota development influenced early childhood growth in preterm infants
Source: Front Pediatr. 2022 Aug 9;10:850629. doi: 10.3389/fped.2022.850629 (PMC9395978; doi:10.3389/fped.2022.850629)

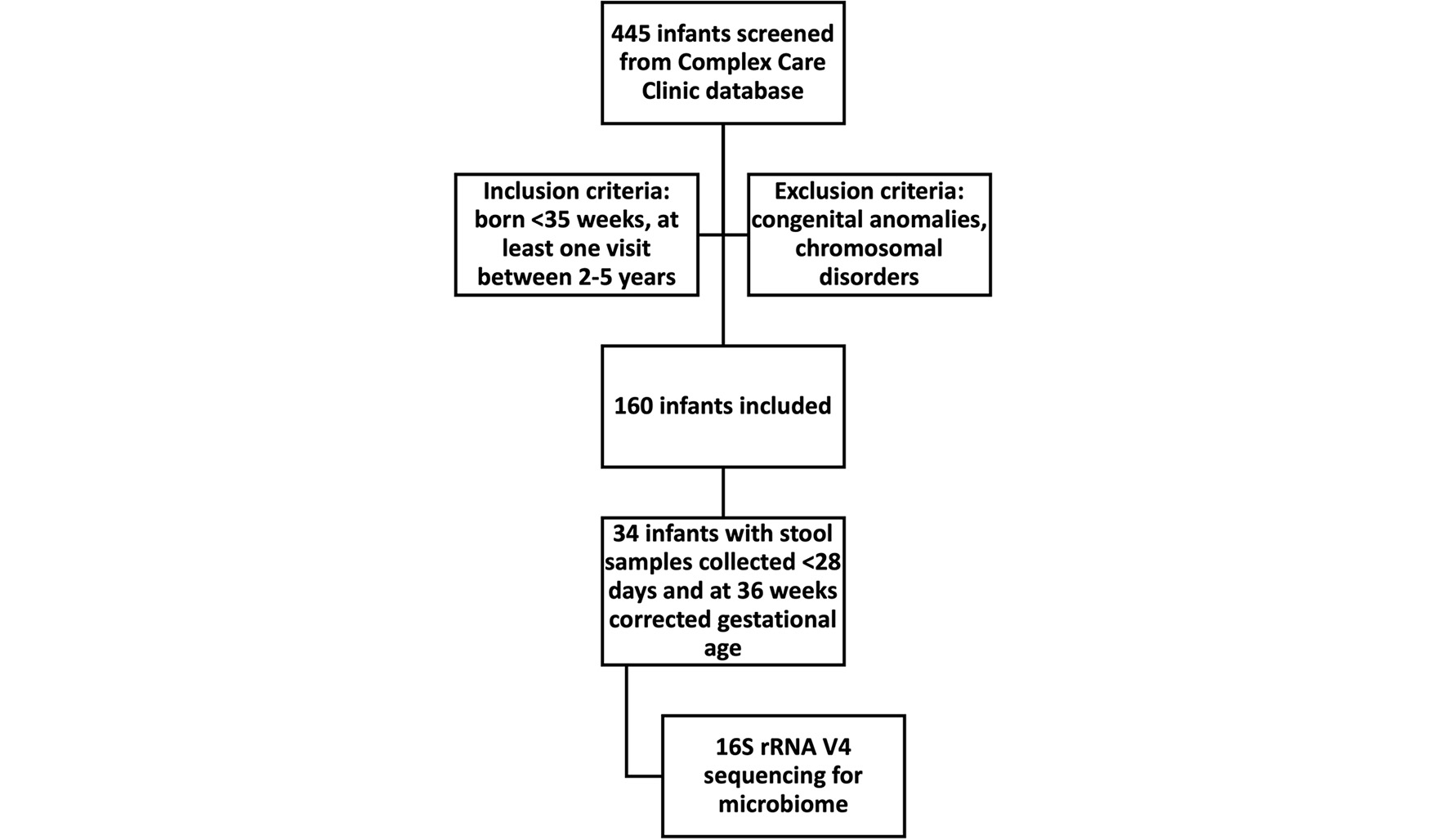

Supplement: Supplementary file 1 [file Image_1.JPEG]
